# Acceptability and practicality of a Spanish translation of paediatric Gait Arms Legs and Spine (pGALS) in Peruvian children

**DOI:** 10.1186/1546-0096-12-48

**Published:** 2014-11-20

**Authors:** Katrina Abernethy, Sharmila Jandial, Lucy Hill, Ernesto Salazar Sánchez, Helen Foster

**Affiliations:** Musculoskeletal Research Group, Institute of Cellular Medicine, Newcastle University, Newcastle, UK; Great North Children’s Hospital, Newcastle Hospitals NHS Foundation Trust, Newcastle, UK; School of Modern Languages, Newcastle University, Newcastle, UK; Hospital Regional de Loreto, Iquitos, Peru

**Keywords:** pGALS, Musculoskeletal, Clinical skills, Medical education, Child, Spanish, Paediatrics

## Abstract

**Background:**

The paediatric Gait, Arms, Legs and Spine (pGALS) musculoskeletal examination tool is validated for use in school-aged English Speaking children and shown to be practical and effective in acute paediatric practice in the UK and Malawi. Our aim was to assess the acceptability and practicality of a Spanish translation of pGALS in an acute paediatric setting in Peru.

**Findings:**

Fifty-three school-aged children presenting to Hospital Regional de Loreto, Peru were recruited to undergo a pGALS examination using a Spanish translation of the instructions. The pGALS examination was completed in 92.5% (49/53), with the time taken (median 4.42 minutes) being acceptable to most parents (98.1%, 52/53). Most children (88.7%, 47/53), found the pGALS examination caused ‘little’ or ‘no additional discomfort’. Using pGALS, significant findings were observed in 18/53 (34%) children; these related to fractures (4/18), hypermobility (4/18), infectious causes (5/18) and soft tissue trauma (5/18).

**Conclusion:**

Using this Spanish translation, pGALS assessment was practical, acceptable and effective in detecting musculoskeletal changes in many children.

**Electronic supplementary material:**

The online version of this article (doi:10.1186/1546-0096-12-48) contains supplementary material, which is available to authorized users.

## Findings

### Background

Paediatric Gait, Arms, Legs and Spine (pGALS) is a simple and quick musculoskeletal (MSK) examination tool, validated in school aged children (4-16 years), with good sensitivity and specificity to detect significant abnormalities [1, 2]. pGALS performs well in the hands of medical students, allowing them to discern abnormal joints in a mean time of 4.25 minutes [3]. Furthermore, pGALS has been shown to be practical and useful when used by non specialists in paediatric MSK medicine in acute paediatric settings [4, 5]. Our aim was to assess the acceptability and practicality of using a Spanish translation of pGALS in acute paediatric setting in Peru.

## Method

The methods used are similar to those used previously [4, 5] to assess acceptability and practicality of pGALS in acute settings. The setting for this study was Hospital Regional de Loreto (HRL), a tertiary hospital, in the Amazon basin of Peru, providing specialist and general paediatric services; HRL has a 60 paediatric ward and typically over 20 children (4-16 years) attending the emergency department per day.

Inclusion criteria for the study included children (4-16 years), both those attending the emergency department and inpatients for any healthcare reason, deemed well enough by doctors and parents to participate. Children and their parent/guardian were approached to take part after routine clinical assessment by the attending doctor. Over a two-week period in 2013, all eligible inpatients and newly presenting children to the emergency department were invited to take part in the study. A proforma was used to collect data including patient demographics, presenting complaint, final diagnosis (from medical case notes). Details of pGALS assessment including abnormalities detected, manoeuvres not completed and the time taken, were recorded. Acceptability in terms of time taken and any additional discomfort caused were assessed using Visual Analogue scales consisting of ‘smiley faces’ [6], and collated from children and parent/guardian.

Informed consent was obtained from the parent/guardian using information sheets and consented forms available in Spanish. A medical student (KA) who is a native English speaker with Spanish as a second language, conducted all communication with families in Spanish and performed pGALS; she had received ‘standard’ pGALS teaching at Newcastle University (a video demonstration of pGALS in a healthy child [7] within a 1 hour seminar delivered by the paediatric rheumatology team).

The pGALS instructions [8] were translated from English into Spanish in collaboration with the School of Modern Languages at Newcastle University. The translated version was discussed with clinicians working in Peru, and no further adjustments were proposed. Ethical approval for the study was obtained from HRL Ethics Committee. Statistical analysis performed using Microsoft Excel and Graphpad Prism 6 software.

## Results

Fifty-three children were recruited (21/53, 39.6% female, 32/53, 60.4% male), with a median age of 9 years (range 4-15 years). Many (29/53, 54.3%) children were assessed in the emergency setting and the remainder (24/53, 45.3%) assessed as inpatients on the ward. The majority (39/53, 73.6%) had infection-related diagnoses with 17/39 (32.1%) having dengue fever. Most (49/53, 92.5%) children completed the entire pGALS examination; gait and leg movements were not completed with reasons including vomiting due to dengue fever (n = 1), limb fracture (n = 2) and snake-bite related pain (n = 1).

Many children (18/53, 34.0%) had an abnormal pGALS assessment with similar rates of abnormalities detected between inpatients (9/24, 37.5%) and emergencies (9/29, 31%). Abnormalities detected (Table 1) included those related to MSK diagnoses (8/53,15.1% - fractures (n = 4) and hypermobility (n = 4)), and to non-MSK diagnoses (10/53, 18.9% - mostly infection and soft tissue trauma).

**Table 1:** **Diagnoses and examination findings in patients with abnormal pGALS examination**

Presenting complaint	Examination findings on pGALS screen	Diagnosis
**Patients with abnormal pGALS findings and MSK diagnoses**		
Limp, painful right leg	Unable to complete leg movements – pain	Fractured femur
	Abnormal appearance of gait and leg	
Swollen elbow, painful left arm	Pain on all arm movements, swelling of elbow	Fractured humerus
Cough, fever	Loss of foot arches, joint hypermobility	Hypermobility/HIV positive, Tuberculosis, pneumonia
Limp, painful leg	Antalgic gait	Tibial diaphysis fracture
	Unable to complete knee and hip movements – pain	
Painful throat and fever	Loss of foot arches, hypermobility	Hypermobility/Viral tonsillitis
Fever and vomiting	Loss of foot arches, pain, hypermobility	Hypermobility/Dengue Fever
Fall and right arm pain	Pain and restriction of all arm movements	Fracture of radius and ulna
Fever	Pain, loss of foot arches, hypermobility	Hypermobility/Suspected Dengue fever
**Patients with abnormal pGALS findings and non-MSK diagnoses**		
Penetrating injury to left foot	Abnormal antalgic gait	Soft tissue trauma
Animal trap trauma to foot	Abnormal gait	Extensive soft tissue injury to right foot
Traumatic injury to chest	Pain on shoulder flexion	Traumatic injury to chest and abdomen, soft tissue injury and liver
Snake bite, painful, swollen left leg	Unable to complete gait examination, pain on knee movements	Snake Bite
Painful, swollen right leg	Leg swelling, abnormal gait appearance	Cellulitis
Facial pain	Pain and restricted movement of Temporomandibular joint (TMJ)	Mastoiditis
Fever, sore throat	Pain on extension of cervical spine	Tonsillitis
Fever, testicular swelling	Antalgic gait	Dengue fever - arthralgia, testicular oedema
	Pain on extension of cervical spine	
Fever and parotid swelling	Pain and restriction at TMJ	Parotid abscess
Traumatic amputation of right hand first finger	Pain on metacarpophalangeal squeeze	Traumatic amputation of right hand first finger
	Pain on finger and thumb movements	

Many children (22/53,41.5%) answered positively to ≥1 of the three pGALS screening questions and of these, 14/22 (63.6%) had joint abnormalities detected. Validity of a positive response to ≥1 question against the pGALS examination findings being abnormal included sensitivity 63.6%, specificity 87.1%, positive predictive value 77.8%, and negative predictive value 77.1%. In terms of correlating abnormalities on pGALS examination, of the three questions, difficulty in dressing was most sensitive in (85.6%), followed by climbing stairs (75%) and pain (63.6%). Four children answered ‘No’ to all three questions but had significant findings on pGALS which included hypermobility (n = 3), jaw movement restriction (n = 1) due to mastoiditis.

**Figure 1:**
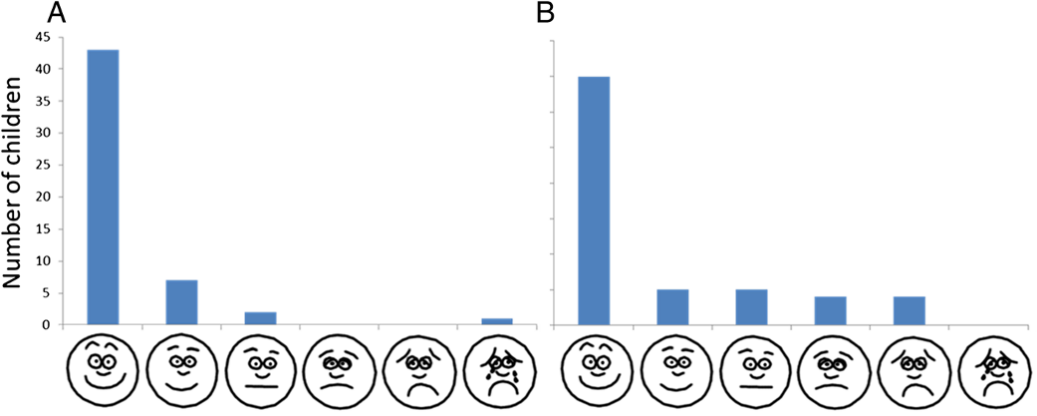
**Acceptability of pGALS - time taken (A) any additional discomfort (B).**

All participants completed the acceptability questionnaire (Figure 1); most (52/53, 98.1%) parents deemed the time taken to be ‘acceptable’ (median time 4.42 minutes, range 2.47-6.50), and the majority of children 47/53 (88.7%), reported that the pGALS examination caused ‘little’ or ‘no discomfort’; of those reporting some additional discomfort, 4/6 (66%) had abnormalities detected (Table 2). Where significant joint findings were found, the time taken for pGALS was significantly longer in comparison with the examination being normal 4.61 minutes (range 3.25-6.20) versus 3.96 minutes (range 2.47 – 6.50), Mann-Whitney *P* =0.038.

**Table 2:** **pGALS assessment findings and diagnoses of children reporting ‘additional discomfort’ during pGALS examination**

Face selected	Number of children	Normal assessment	Diagnoses	Abnormal assessment	Diagnoses
	3	2	Dengue fever (n = 2)	1	Fracture right humerus (n = 1)
	3	0		3	Snake bite (n = 1)
					Parotid abscess (n = 1)
					Extensive soft tissue injury to foot (n = 1)
	0	0		0	

## Discussion

This is the first study, to our knowledge, to describe a Spanish version of pGALS and show that it is practical and acceptable to children and parents in an acute paediatric setting. Furthermore we have demonstrated that pGALS detects abnormalities in many children similar to previous studies [4, 5].

The Spanish translation of pGALS [Additional file 1] was a forward translation (English to Spanish) of the instructions for the manoeuvres of pGALS. Whilst using pGALS, the verbal instructions in Spanish were supplemented by a ‘copy me’ approach by the examiner (KA) as recommended in our teaching [2] and this may well have facilitated understanding. Nonetheless we believe the Spanish translation of pGALS to be valid as exemplified by no further amendments being deemed necessary by native Spanish clinicians at HRL, and all children being able to perform the manoeuvres appropriately. We acknowledge that the findings of pGALS by the medical student were not validated by MSK ‘experts’ (e.g. paediatric rheumatologists or orthopaedic surgeons) but we regard the findings useful to assess practicality and acceptability rather than the validity of pGALS per se.

Many children were able to complete the full pGALS assessment and acceptability was high with no difference in the completion rates encountered between the emergency and inpatient settings; this is notable given that many had significant trauma (including fractures) or serious infections (such as malaria and dengue fever); we acknowledge that children deemed too unwell to take part by the attending paediatrician were not included in the study and hence there was potentially selection bias. Abnormalities were commonly found using pGALS, both in those with, and those without underlying MSK diagnoses; this corroborates previous reports emphasising the need to consider findings within the clinical context [4, 5]. The mean time for performing pGALS was similar to that previously described by medical students [3] and not surprisingly, was longer than the time taken by paediatric rheumatologists or primary care doctors to perform pGALS [1, 4, 5].

In this study, the sensitivity of the three pGALS screening questions was higher than reported previously (sensitivity 63.6%, specificity 87.1%) [1, 4, 5], with many children reporting positive responses for pain, problems with function including dressing and undressing or difficulties on stairs. In contrast to the similar study of pGALS used in Malawi [5], where few children are exposed to stairs, the screening questions are culturally relevant in this region of Peru with houses commonly having interior or exterior stairs to protect from ground level flooding [5]. The importance of MSK examination, even in the absence of a positive response to the questions, is emphasised by some children having significant joint changes albeit denying pain or functional problems; this corroborates previous work showing that the history alone is insufficient [9]. Len et al [10] developed a useful screening questionnaire for Juvenile Idiopathic Arthritis to be completed by parents in a Latin American population; whilst this questionnaire was shown to be useful as a screening tool, the pGALS examination has the added advantage of identifying joint changes that are not overtly symptomatic and may be missed if there is reliance on a questionnaire per se. Potentially therefore the synergistic role of both tools in clinical practice to identify children with significant MSK problems could be further investigated and emphasises the importance of both targeted history taking and physical examination.

We have shown that pGALS is practical, acceptable and useful in the assessment of acutely unwell children in this setting. Furthermore we highlight the importance of MSK assessment as part of general paediatrics. The Spanish translation of pGALS appeared effective and the screening questions were culturally relevant. It is hoped that the availability of the Spanish translation will further increase the uptake of pGALS in clinical practice, facilitate improved recognition of MSK problems and ultimately improve clinical care.

## Electronic supplementary material

Additional file 1: **Spanish translation of pGALS.** (PDF 653 KB)

## Abbreviations

MSK: Musculoskeletal
pGALS: paediatric Gait, Arms, Legs and Spine
HRL: Hospital Regional de Loreto.
