# Supplementary material for: Acceptability and practicality of a Spanish translation of paediatric Gait Arms Legs and Spine (pGALS) in Peruvian children
Source: Pediatr Rheumatol Online J. 2014 Nov 20;12:48. doi: 10.1186/1546-0096-12-48 (PMC4395967; doi:10.1186/1546-0096-12-48)
Supplement: Supplementary file 1 — Additional file 1: Spanish translation of pGALS. (PDF 653 KB) [file 12969_2014_2182_MOESM1_ESM.pdf]

Spanish translation of pGALS

Un examen pGALS, es un examen utilizado por los médicos y otros profesionales de la salud para detectar anomalías del aparato locomotor y la discapacidad funcional en relación con la manera de andar, los brazos, las piernas y la columna vertebral.

Preguntas de detección.

- ¿Tienes (o tiene tu hijo) dolor o rigidez en las articulaciones, los músculos o la espalda?
- ¿Tienes (o tiene tu hijo) dificultad para vestirse sin ayuda?
- ¿Tienes (o tiene tu hijo) problemas para subir y bajar las escaleras?

	**Maniobras de detección**	**Lo que se está evaluando**
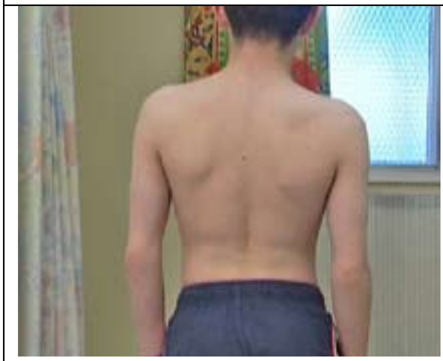	Observe al niño parado. (De frente, de espalda, y de lado)	• Postura y habito corporal<br>• Erupciones en la piel,<br>• Deformidad – como discrepancia entre la longitud de las piernas, alineación de las piernas, escoliosis, hinchazón de los articulaciones, desgaste muscular, pies planos
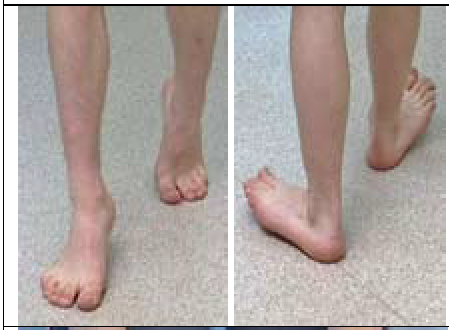	Observe al niño caminando.<br><br>“Camina en los talones”<br><br>“Camina en la punta de los dedos”	• Tobillos, articulaciones del pie,<br>• Postura del pie y la presencia de los arcos del pie.
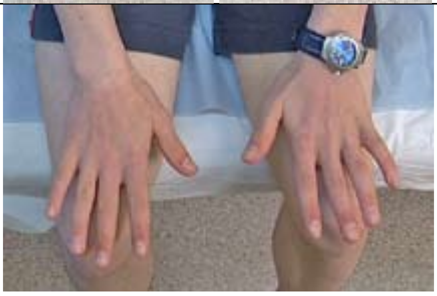	“Estira las manos enfrente tuyo”	• Flexión anterior de los hombros,<br>• Extensión de los codos<br>• Extensión de las muñecas<br>• Extensión de los articulaciones pequeños de los dedos

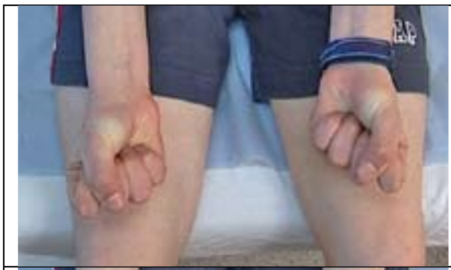	“Voltea las manos hacia arriba y haz un puño”	• Supinación de las muñecas<br>• Supinación de los codos<br>• Flexión de los articulaciones pequeños de los dedos.
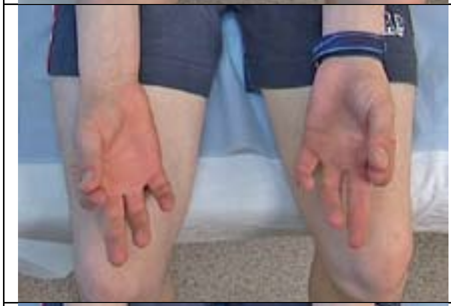	“Junta el dedo pulgar con el dedo índice”	• Destreza manual<br>• Coordinación de las articulaciones pequeñas del dedo índice y del pulgar.
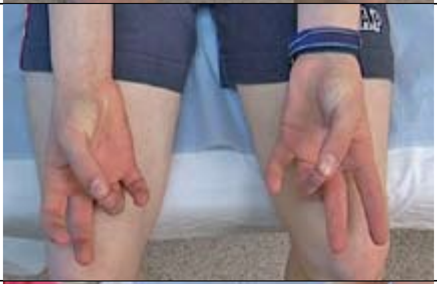	“Tócate las puntas de los dedos”	• Destreza manual<br>• Coordinación de las articulaciones pequeñas de los dedos y del pulgar.
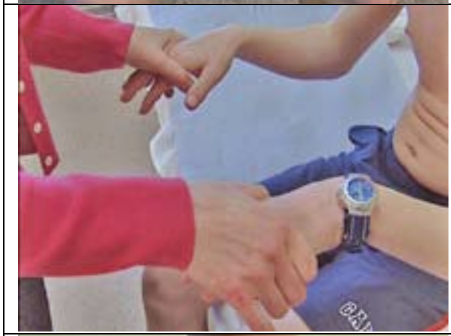	Apriete las articulaciones entre los metacarpos y las falanges para evaluar la sensibilidad.	• Las articulaciones entre los metacarpos y las falanges
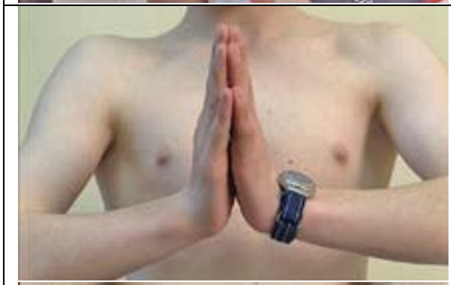<br>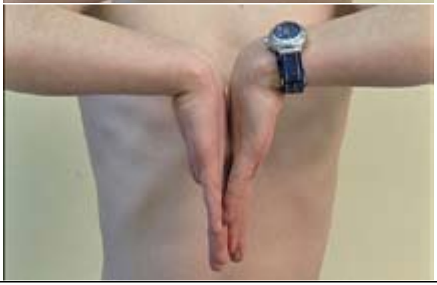	“Pon las manos juntas palma con palma”<br>“Pon las manos dorso con dorso”	• Extensión de las articulaciones pequeñas de los dedos<br>• Extensión de las muñecas<br>• Flexión de los codos.

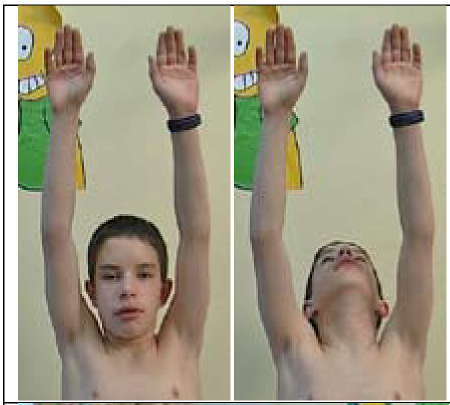	“Estira las manos hacia arriba”, “toca el cielo” y “mira el techo”	• Extensión de los codos<br>• Extensión de las muñecas<br>• Abducción de los hombros<br>• Extensión del cuello
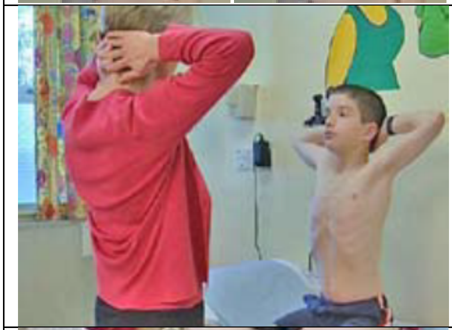	“Coloca las manos por detrás del cuello”	• Abducción de los hombros<br>• Rotación externa de los hombros<br>• Flexión de los codos.
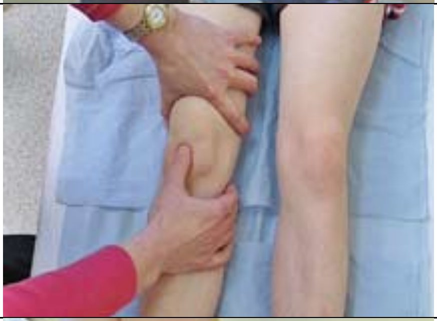	Palpe la rodilla para detectar derrame de fluidos.	• Efusión de la rodilla
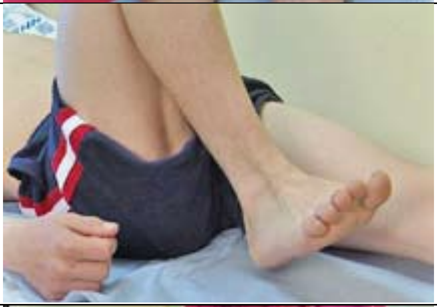	Movimiento activo de las rodillas (flexión y extensión) para detectar crepitaciones o crujidos.	• Flexión de la rodilla<br>• Extensión de la rodilla
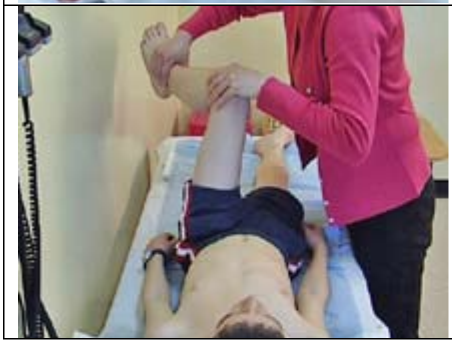	Movimiento pasivo de la cadera (rodilla doblada a 90 grados y rotación interna de cadera)	• Flexión de la cadera<br>• Rotación interna de la cadera

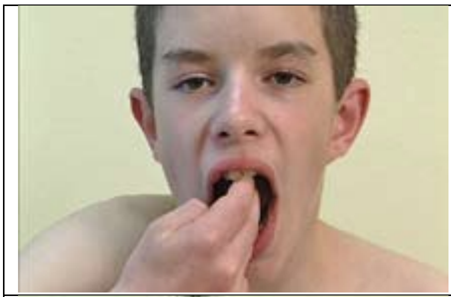	“Abre la boca ampliamente y coloca tres dedos dentro”	• Articulación del temporal y la mandíbula
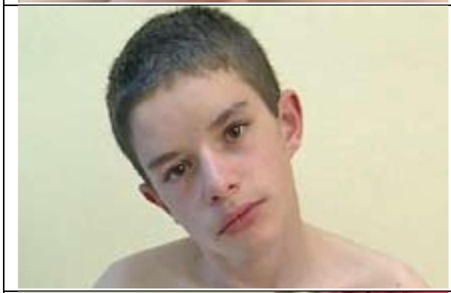	“Junta la oreja con el hombro”	• Columna cervical
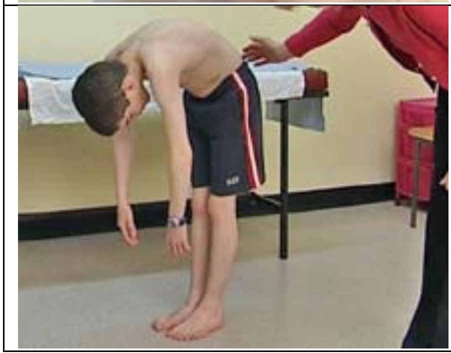	“Inclina el cuerpo hacia adelante y tócate los dedos de los pies”	• Flexión anterior de la columna toracolumbar<br>• Escoliosis

Images reproduced with kind permission of Arthritis Research UK
